# Early recruitment of PARP-dependent m^8^A RNA methylation at DNA lesions is subsequently accompanied by active DNA demethylation

**DOI:** 10.1080/15476286.2022.2139109

**Published:** 2022-11-16

**Authors:** Soňa Legartová, Alena Svobodová Kovaříková, Jana Běhalová Suchánková, Hana Polášek-Sedláčková, Eva Bártová

**Affiliations:** Department of Cell Biology and Epigenetics, Institute of Biophysics of the Czech Academy of Sciences, Královopolská 135, 612 65, Brno, Czech Republic

**Keywords:** DNA repair, epigenetics, RNA methylation, base excision repair, DNA demethylation

## Abstract

RNA methylation, especially 6-methyladenosine (m^6^A)-modified RNAs, plays a specific role in DNA damage response (DDR). Here, we also observe that RNA modified at 8-methyladenosine (m^8^A) is recruited to UVA-damaged chromatin immediately after microirradiation. Interestingly, the level of m^8^A RNA at genomic lesions was reduced after inhibition of histone deacetylases and DNA methyltransferases. It appears in later phases of DNA damage response, accompanied by active DNA demethylation. Also, PARP inhibitor (PARPi), Olaparib, prevented adenosine methylation at microirradiated chromatin. PARPi abrogated not only m^6^A and m^8^A RNA positivity at genomic lesions, but also XRCC1, the factor of base excision repair (BER), did not recognize lesions in DNA. To this effect, Olaparib enhanced the genome-wide level of γH2AX. This histone modification interacted with m^8^A RNAs to a similar extent as m^8^A RNAs with DNA. Pronounced interaction properties we did not observe for m^6^A RNAs and DNA; however, m^6^A RNA interacted with XRCC1 with the highest efficiency, especially in microirradiated cells. Together, we show that the recruitment of m^6^A RNA and m^8^A RNA to DNA lesions is PARP dependent. We suggest that modified RNAs likely play a role in the BER mechanism accompanied by active DNA demethylation. In this process, γH2AX stabilizes m^6^A/m^8^A-positive RNA-DNA hybrid loops via its interaction with m^8^A RNAs. R-loops could represent basic three-stranded structures recognized by PARP-dependent non-canonical m^6^A/m^8^A-mediated DNA repair pathway.

## Introduction

Cells developed sophisticated mechanisms for maintaining genome integrity. Genome injury leads to single-strand breaks (SSBs) or more deleterious double-strand breaks (DSBs) in DNA. In a simplistic view, three repair mechanisms recognize single-strand damage in DNA. Base excision repair (BER) eliminates damaged bases by generating SSBs via the function of DNA glycosylases. The next step of DNA repair is mediated by DNA polymerase β (polβ). This enzyme is recruited to DNA lesions by PARP1 (poly[ADP-ribose] polymerase 1), but subsequently, the XRCC1 protein is responsible for PARP1 release from SSBs^1^. During long-patch BER, together with PCNA, pol δ and pol ε mediate DNA synthesis 2. After that, DNA ligases are responsible for the final step of the BER mechanism. For example, DNA ligase IIIα, in parallel with XRCC1, catalyzes the so-called short-patch BER in human cells [3]. On the other hand, DNA ligase I plays a role in long-patch BER [4]. The second mechanism, nucleotide excision repair (NER), recognizes UV-induced DNA lesions. Such molecular lesions are mainly cyclobutane pyrimidine dimers (CPDs) or 6–4 photoproducts. In this case, the DNA polymerase uses an undamaged DNA strand as a template for synthesizing the damaged strand. NER is distinguished as Global Genomic NER or Transcription Coupled NER. It is well known that many proteins function in the NER pathway, including XPA, XPB, XPC, XPD, XPE, XPF, and XPG, CSA, CSB, RASD23A, RAD23B or others [5]. The third SSB repair mechanism is mismatch repair (MMR) which recognizes mismatches in bases or insertion/deletion mispairs that appear during DNA replication and recombination 6.

The most deleterious are so-called double-strand breaks (DSBs), in which incorrect repair leads to tumorigenesis. To avoid pathological processes caused by non-physiological repair of DSBs, the following two canonical and mechanistically distinct repair pathways are initiated: Non-homologous end joining (NHEJ), active mainly in the G1 phase of the cell cycle, and less erroneous homologous recombination repair (HRR) that needs nascent chromatids and, thus, the entry of the cells to the S phase of the cell cycle. It is well known that the HRR also proceeds in the G2 phase of the cell cycle [7]. In the S/G2 phases, the NHEJ repair mechanism can also work, but only when the HRR process fails in pathological states 8. In a physiological condition, 53BP1 (p53-binding protein 1) and its interacting partner RIF1, proteins of NHEJ, form a barrier inhibiting DNA-end resection that is, on the other hand, activated by the BRCA1 protein interacting with CtIP in a phospho-dependent manner [9,10]. From the view of NHEJ repair machinery, Ku proteins (Ku70 and Ku80) are essential factors that recognize asymmetric DSBs and recruit DNA protein kinase catalytic subunits (DNA-PKcs). This enzyme phosphorylates the Artemis protein having a 5’-3’ exonuclease activity. This process leads to the formation of blunt DSBs. After that, DNA ligase joins two strands of DNA that lost original genetic information. On the other hand, more sequentially precise HRR needs a 3’ overhang and subsequent synthesis of a complementary strand according to the sister chromatid template or following chromatid of the homologous chromosome. As an early step of HRR is considered the binding of the MRN (Mre11/Rad50/NBS1) protein complex to asymmetric double-strand break sites containing 3’ overhangs that are generated by nucleolytic degradation of the 5’ strands. In this case, also RPA covers 3’ overhangs to protect DNA against nucleases. Such single-strand DNA nucleofilaments are recognized by the RAD51 protein that recruits the BRCA2 protein. This protein complex replaces RPA. In this DNA repair process, RAD51 helps to search for homologous DNA and mediates the formation of so-called D-loops. This step needs a homologous DNA template. In this case, non-cross-over HRR or cross-over HRR can be recognized [11].

DNA repair machinery has its epigenetic regulations. It is generally accepted that epigenetic adaptations of DNA repair systems involve the function of phosphorylated histones H2AX (γH2AX). Also, histone H4 lysine 20 methylation (H4K20me2/me3) or H3K79 methylation, as prominent binding partners of the 53BP1 protein [12,13], represent crucial repair factors at DSB sites in mammalian cells [14–18]. From the epigenetic point of view, ionizing radiation causes changes in H3K36 methylation and H3K9me3, which represent additional epigenetic factors of DDR [19–24]. It is known that H3K9me3 at damaged chromatin is linked to the activation of histone acetyltransferase TIP60, furthermore mediating specific histone acetylation at DNA lesions [23,24]. Also, H2AXK119 ubiquitination and H4K16 acetylation were observed at damaged chromatin. Similarly, the NuRD complex, consisting of histone deacetylases 1 and 2 (HDAC1 and HDAC2), is recruited to the damaged genome. This epigenetic event causes deacetylation of H3K56 [25]. We additionally observed H3K9 deacetylation at microirradiated chromatin. This phenomenon was likely mediated by HDAC1 that significantly accumulated at DSB sites [26].

It was recently published that histone signature and co-transcriptionally modified RNAs participate in DNA damage response. It was shown that a novel non-canonical repair pathway is mediated via RNAs methylated on N^6^ -adenosine (m^6^A) [27,28]. It is well known that this RNA modification is regulated by specific ‘writers’, so-called methyltransferases METTL3, METTL14, and METTL16, and recognized by ‘readers’ like the YTHDC1 protein. As fundamental m^6^A ‘erasers’ are considered FTO (fat mass and obesity-associated protein) and ALKBH5 proteins (summarized by [29]). Recently, we additionally showed that mainly METTL16 is recruited to UVA-microirradiated chromatin in later steps of DDR, while the levels of METTL3 and METTL14 proteins remain stable at DNA lesions [27]. Xiang et al. (2017) [28] published that N^6^-methyladenosinepresent on RNA appears at DNA lesions immediately after local laser microirradiation, and we documented that this DDR-related event is additionally accompanied by depletion of 2,2,7-methylguanosine (m_3_G/TMG) in RNA [27]. Significantly, in parallel with the described changes in epitranscriptome, UV-irradiation additionally decreases the global cellular level of N^1^-methyladenosine (m^1^A) in RNAs [27]. In this case, surprisingly, when m^6^A RNAs disappeared from DNA lesions, the level of FTO remained stable [27]. The above-mentioned experimental data documents that the function of m^6^A-specific ‘writers’ and ‘erasers’ is essential for the regulation of not only gene expression but also for DNA damage repair. For instance, it was shown that in the absence of METTL3, there is a delay in the repair of UV-induced cyclobutane pyrimidine dimers (CPDs), and cells are more sensitive to UV light [28,30]. It is also well known that DNA polymerases participate in such DNA damage responses. In this case, DNA polymerase κ (Pol κ), playing a role in nucleotide excision repair (NER), works in parallel with the catalytic activity of METTL3 recruited to damaged chromatin. Xiang et al. (2017) [28] showed that exogenous overexpression of DNA Pol κ rescues the defect of CPD elimination in METTL-depleted cells. This observation suggests that the fast recruitment of DNA Pol κ to the damage site is potentially due to m^6^A RNA deposition at UV-irradiated genomic regions [30]. Xiang et al. (2017) [28] suggested that METTL3/METTL14 complex (but not METTL3/WTAP complex) in parallel with FTO (but not ALKBH5) serves as the ‘writer’ and the ‘eraser’ of the m^6^A in RNA accumulating at UV-damaged chromatin. Zhang et al. (2020) [31] showed that ATM-mediated phosphorylation at serine 43 could activate METTL3, and such phosphorylated protein accumulates at DNA lesions, where it acts as methyltransferase mediating the m^6^A in RNA. Also, m^6^A ‘reader’, the YTHDC1 protein, is recruited to damaged sites [31]. These all observations indicate the existence of a non-canonical m^6^A-mediated DNA repair pathway. It was revealed that this non-canonical process is dependent on the PARP [Poly (ADP-ribose) polymerase] and is specific for UV-irradiated chromatin but not for ionizing-radiation induced foci (IRIF) [28,29,32–37]. Zhang (2017) [37] suggested that the m^6^A mediated DNA repair pathway is independent of the phosphorylation of histone H2AX and is activated at damage sites with hybrid DNA-RNA loops. It was published that such RNA-loops appear in the genome because of DNA damage at transcription sites, and some studies showed their link to the Transcription-Coupled Nucleotide Excision Repair mechanism (TC-NER) [38].

We were inspired by the above-mentioned studies showing novel and RNA-dependent DNA repair principles. To continue with the study of the role of RNA modifications in DNA damage response, we addressed whether RNA methylated at 8-adenosine (m^8^A RNA) can recognize UV-induced DNA lesions. This RNA modification is known to be catalysed by the SAM (S-Adenosyl methionine)-dependent methyltransferase Cfr [39]. The studies were performed in bacteria, especially in *Escherichia coli*, in which the Cfr methyltransferase is responsible for the resistance to five different classes of antibiotics. It was described that the Cfr-mediated modification was determined on nucleotide A2503 of 23S rRNA, and also Cfr can catalyse methylation leading to the formation of 2,8-dimethyladenosine. However, the mutation of single conserved cysteine residues in the SAM motif CxxxCxxC of Cfr abolishes its activity [39].

Here, we show m^8^A RNA positivity at DNA lesions experimentally induced in human cells. The local high positivity of m^8^A RNA at UV-damaged chromatin depended on the PARP function. It was shown by other authors that PARP inhibitors trap the PARP1 and PARP2 enzymes in damaged DNA. Such PARP-DNA complexes are more likely to be cytotoxic than unrepaired SSBs induced by PARP inhibitors themselves [1,40]. Since the presence of PARP1 at damaged chromatin suppresses the activities of BER factors, including APE1, Polβ, and LigIIIα, it seems likely that PARP1 function is preferentially linked to the BER mechanism [41,42]. However, an indirect role of PARP1 in BER was shown by Strom et al. (2011) [43], suggesting that indirect regulation of the DNA repair process is via auto PARylation of PARP1 and PARP2 or PARylation of various chromatin-related proteins that facilitate the recruitment of DNA repair factors [44]. From this view, we show the PARP-dependent appearance of m^8^A RNAs at DNA lesions, and we also discuss the existence of an RNA-dependent non-canonical DNA repair pathway involving both m^6^A RNAs [45] and RNAs methylated on 8-adenosine.

## Results

### The 8-methyladenosine (m^8^A)-modified RNA is recruited to UVA-microirradiated chromatin

**Figure 1.**
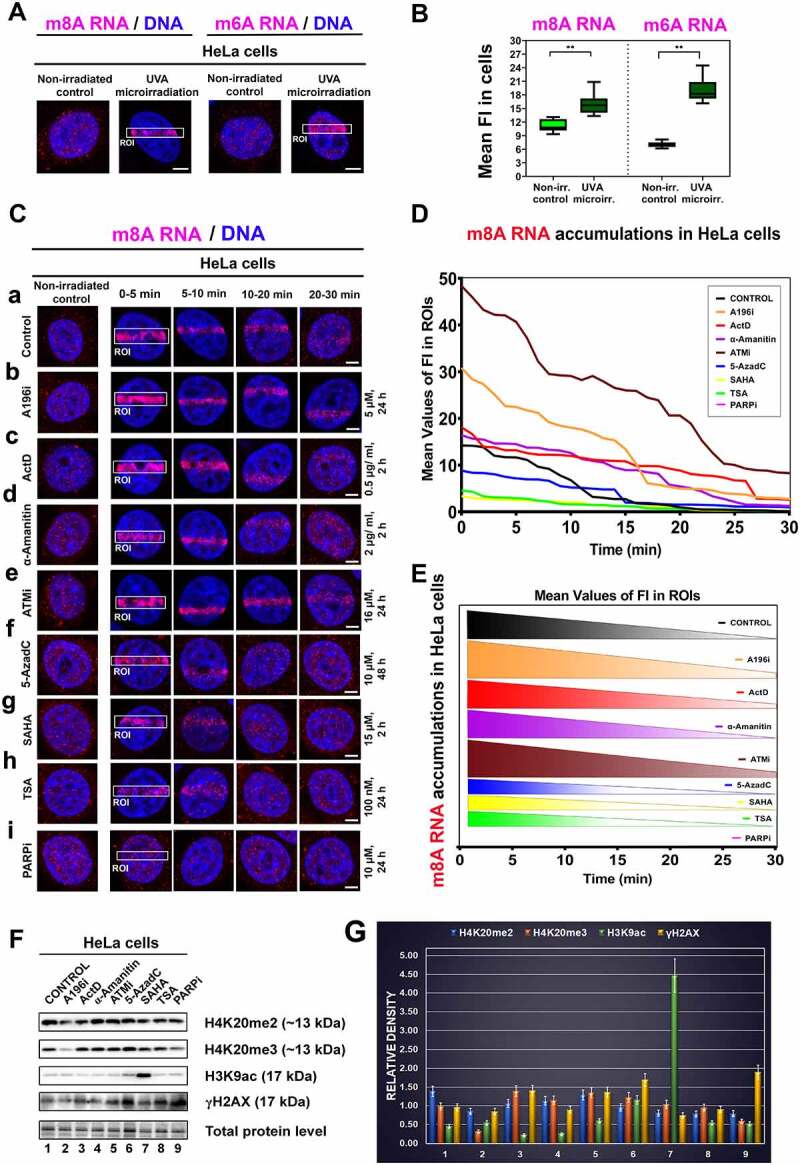
Recruitment of m^6^A RNA and m^8^A RNA to locally irradiated chromatin. (A) Localization of m^6^A RNA and m^8^A RNA in the nucleoplasm of non-irradiated and microirradiated HeLa cells. (B) Quantification of the density of m^6^A RNA and m^8^A RNA in the whole cell nuclei of non-irradiated and microirradiated cells. (C) m^8^A RNA was studied at DNA lesions of (a) non-treated cells, (b) after the treatment of Suv20h1/2 inhibitor A196 (A196i) affectioning H4K20me2/me3, after inhibition of RNA polymerases I and II (c) Actinomycin D and (D) α-amanitin. (E) We performed ATM depletion and (F) inhibition of DNA methyltransferase by 5-aza deoxycytidine. Inhibitors of histone deacetylases (HDACs) (G), suberoylanilide hydroxamic acid (Vorinostat; syn. SAHA), and (H) Trichostatin A (TSA) were studied. (I) PARP was inhibited by Olaparib. Regions of interest (ROI) are shown in white frames. Scale bars show 5 µm. (D, E) Quantification of m^8^A RNA at locally microirradiated chromatin in non-treated cells and after different treatments is shown. **(F)** Western blots show effects of selected treatments from panel C on the level of H4K20me2/me3, H3K9ac, and γH2AX. Data were normalized to the total protein levels and quantification is shown in panel **(G).**

We analysed whether m^6^A RNA [26–28] and m^8^A RNA are recruited to UV-induced DNA lesions. As the first step, we studied the distribution properties of m^6^A RNA and m^8^A RNA in the nucleoplasm. We observed a lower level of both RNA modifications in the nucleoplasm of non-irradiated cells, while the density of m^6^A RNAs and m^8^A RNAs, measured in the whole cell nuclei, was higher after laser microirradiation. The highest m^6^A RNA and m^8^A RNA levels were found in microirradiated genomic region. These data implied that modified RNA is not recruited from the surrounding genome to damaged sites, but RNA is directly methylated at DNA lesions (Fig. 1A,). After initiation experiments, we studied the recruitment kinetics of m^8^A RNAs to DNA lesions. We observed that m^8^A RNAs recognize micro-irradiated chromatin immediately after local laser exposure. However, m^8^A RNAs’ positivity weakens 15–30 minutes after microirradiation (Fig. 1C-E). As the next step, we analysed if distinct inhibitors of epigenetic processes and DNA repair can change the recruitment properties of ^8^A RNAs to DNA lesions. We studied the effect of Suv420H1 and Suv420H2 inhibitor A196 (A196i[46]) affectioning H4K20me2/me3, a prominent 53BP1 binding partner playing a role in the NHEJ repair mechanism [18,47] (Fig. 1C-E). In the case of histone signature, replication must be considered from the view of the dilution of the histone marks, including H4K20me2/me3. As a result, a reduced level of the 53BP1 protein, as an H4K20me3 binding partner, at DNA lesions is expected in the S/G2 phases of the cell cycle. However, this was not the case for the 53BP1 protein because we recently showed a comparable level of 53BP1 at microirradiated chromatin of cells studied in the G1, S, and G2 cell cycle phases [48]. These data show that a subset of old and likely modified histones is deposited into newly-synthesized chromatin after replication. Alternatively, as discussed by Budhavapu et al. [49] rather, histone-modifying enzymes, but not the parental histones, represent epigenetic factors that remain associated with the DNA through replication. In this case, these enzymes can re-establish the epigenetic information on the newly assembled chromatin. Interestingly, Svobodova Kovarikova et al. [18] showed a reduced level of H4K20me3 at UV-induced DNA lesions in the S/G2 cell cycle phases, so we might expect a reduced level of 53BP1 at microirradiated chromatin in G2 cells, but this was not that case [48]. Moreover, the 53BP1 protein can bind to γH2AX, which is also a 53BP1 interacting partner; thus, not only H4K20me2/me3 but also γH2AX can be a key factor in this DNA repair mechanism (summarized in [50]).

We also addressed how the following compounds affect m^8^A RNA positivity at DNA lesions. We analysed the effect of inhibitors of RNA polymerases I and II (Actinomycin D and α-amanitin). We performed ATM inhibition (ATMi), which could affect DDR processes. We also studied the effect of inhibition of DNA methyltransferase by 5-aza deoxycytidine or inhibition of histone deacetylases (HDACs) by Trichostatin A (TSA) or suberoylanilide hydroxamic acid (Vorinostat; syn. SAHA) (Fig. 1Cb-). Moreover, we inhibited PARP with Olaparib for 1 h and 24 hours (Fig. 1Ci, 1-F). In the suggested experiments, quantification analyses showed that ATMi and A196i strengthen the accumulation of m^8^A RNA at locally micro-irradiated chromatin (Fig. 1D,). At the same time, HDAC inhibitors, TSA, and SAHA (inducing hyperacetylation) reduced the level of m^8^A RNAs at locally induced DNA lesions (Fig. 1D,). Significantly, PARP inhibitor Olaparib completely abrogated m^8^A RNA positivity at DNA lesions (Fig. 1Ci). Recruitment kinetics of m^8^A RNA to DNA lesions also changed over time. The most pronounced DDR-related accumulation of m^8^A RNA was 0–5 min after micro-irradiation. The significantly reduced m^8^A RNA signal at DNA lesion was 15–30 min post-irradiation, especially in 5-AzaC, TSA treated the cells, or after SAHA treatment (Fig. 1D). We quantified the density of m^8^A RNA in the microirradiated region in time, and the treatment-dependent differences in this parameter are shown in Fig. 1D,E. Also, we studied the effect of selected inhibitors on histone signature, and we confirmed that A196i reduces the level of H4K20me2, and especially H4K20me3. SAHA caused H3K9 hyperacetylation, and both 5-AzaC and PARPi increased the level of γH2AX; thus, these compounds induced DNA damage (Fig. 1F). Taken together, these experiments imply that especially PARP inhibitor Olaparib and HDAC inhibitors reduce the level of m^8^A RNA at DNA lesions.

### A distinction in the density of m^6^A RNA and m^8^A RNA at DNA lesions is only in the G1 phase of the cell cycle, and m^6^A RNA positivity at damaged chromatin is METTL3-dependent

**Figure 2.**
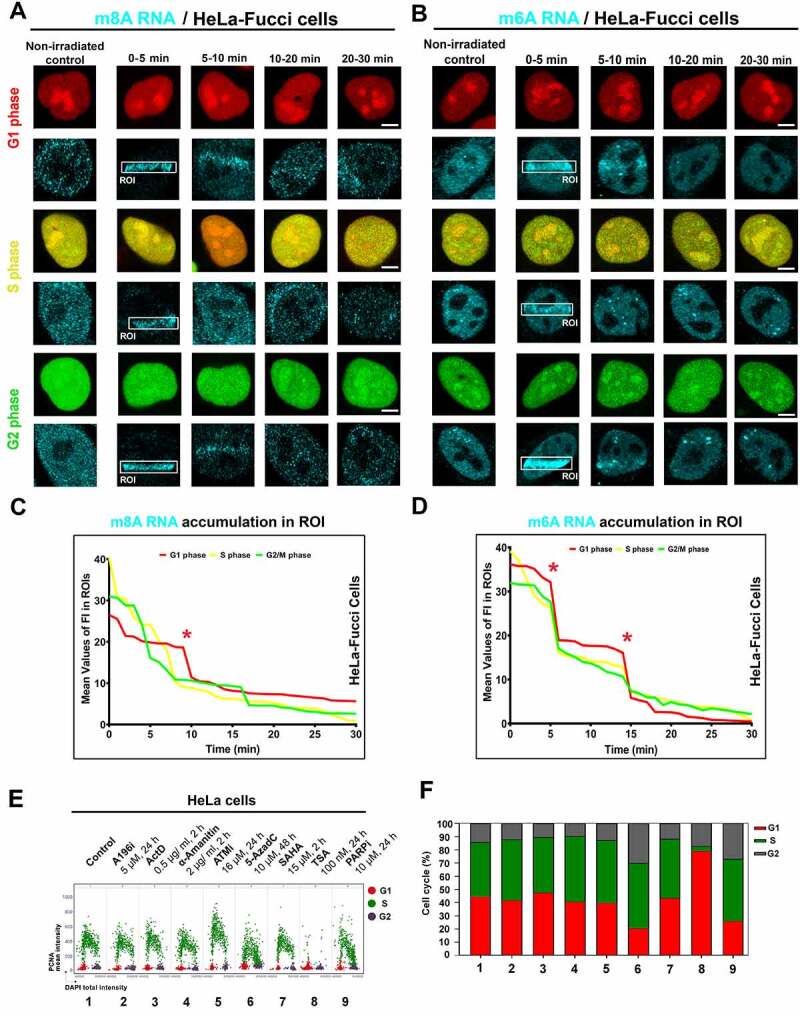
Accumulation of m^8^A RNA and m^6^A RNA to DNA lesion during interphase. Using the FUCCI cellular system expressing RFP-tagged cdt1 in the G1 phase and GFP-tagged geminin in the G2 phase of the cell cycle, we observed an accumulation of both (A) m^8^A RNA and (B) m^6^A RNA to microirradiated chromatin in G1, S, and G2 phases of the cell cycle. Quantification of (C) m^8^A RNA and (D) m^6^A RNA fluorescence intensity in irradiated regions of G1, S, and G2 cells.

To the phenomenon mentioned above, we additionally observed that the recruitment of m^8^A RNA to DNA lesions is stable during the interphase of the cell cycle. It is documented in HeLa cells stably expressing the so-called FUCCI system. It is possible to recognize cells in the G1 phase expressing RFP-tagged cdt1 and G2 phase cells expressing GFP-tagged geminin. The S-phase can be identified according to the weak expression of both markers, see Fig. 2A. Density of m^8^A RNA in irradiated chromatin of FUCCI cells was quantified in Fig. 2C, showing a maximum intensity peak 10 minutes after microirradiation. Similarly, m^6^A RNA recognized locally induced DNA lesions in all cell cycle phases (Fig. 2B,). Notably, a distinction in the density of m^6^A RNA and m^8^A RNA recruitment to DNA lesions was only in the G1 phase of the cell cycle. In detail, m^6^A RNAs had a maximum peak of 5 and 15 minutes at DNA lesions, while the highest level of m^8^A in RNA was in UV-damaged chromatin at 10 minutes postirradiation (Fig. 2C,). We also studied the effect of selected inhibitors on the cell cycle profile. By the use of quantitative microscopy, we observed that especially 5-AzaC, TSA, and PARPi change the cell cycle profile (Fig. 2E,). As mentioned above, the positivity of modified RNAs in DNA lesions was in all cell cycle phases studied (Fig. 2A,); thus, the changes in the cell cycle could not influence the fact that modified RNAs recognize UV-damaged chromatin irrespectively of the cell cycle.

**Figure 3.**
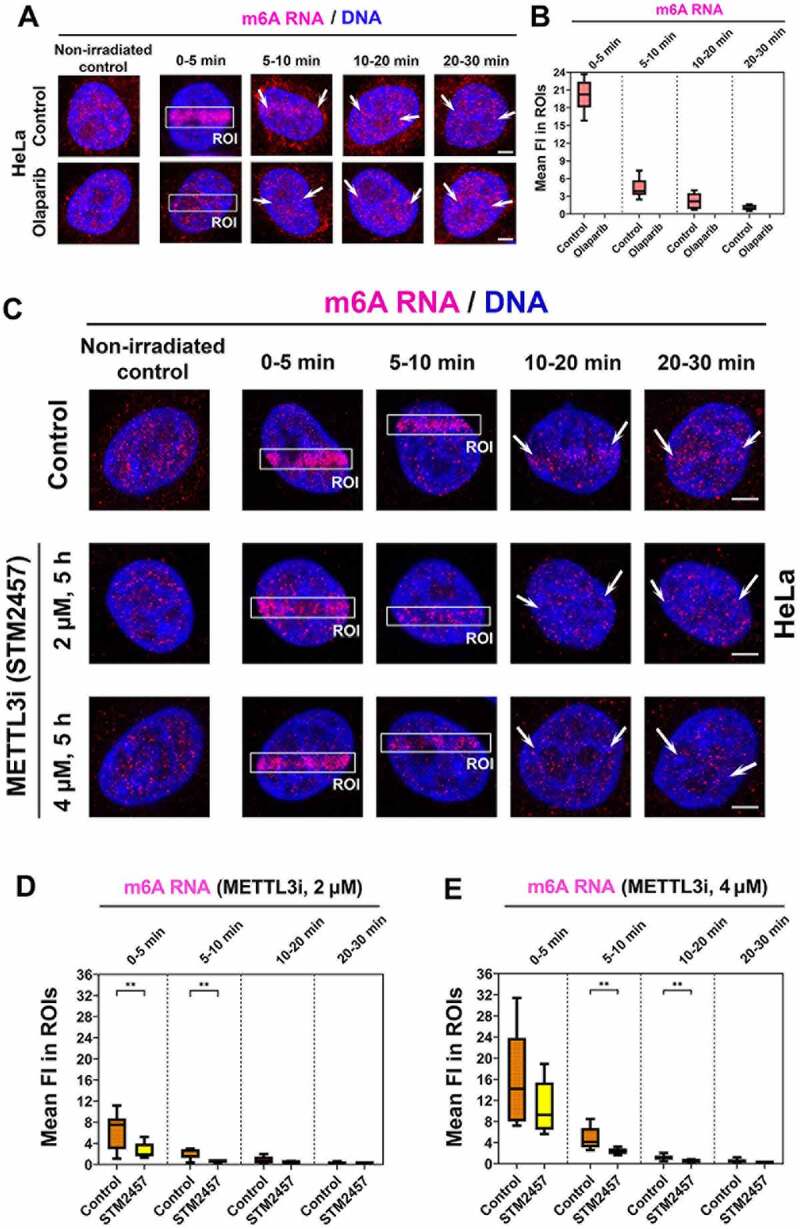
The level of m^6^A RNA in microirradiated regions and the effect of METTL3 inhibitor. (A) m^6^A RNA accumulated in microirradiated chromatin 0–5 min after laser exposure. (A, B) Quantification by ImageJ software shows that PARP inhibitor Olaparib reduced m^6^A RNA positivity at DNA lesions. (C-E) METTL3 inhibitors, STM2457 (see ref[51].), reduced the level of m^6^A RNA at microirradiated chromatin. Regions of interest (ROI) are shown by rectangles or, in later phases of DDR, by arrows for better visibility. Statistically, significant differences are shown at p ≤ 0.01. Scale bars show 5 µm.

In parallel with the kinetics of m^8^A RNA at locally induced DNA lesions after PARP inhibition (Fig. 1 Ci, D, E), we have also studied the kinetics of m^6^A RNA at DNA lesions after PARP inhibition. We came to similar results for both m^6^A RNA and m^8^A RNA, meaning that the PARP inhibitor (Olaparib) abolished the positivity of both m^6^A RNA and m^8^A RNA at DNA lesions (Fig. 1 Ai , and 3A, B). In this case, we suggest that PARPi-induced changes in the cell cycle profile could not influence our conclusion on PARP-dependent and cell cycle-independent recruitment of modified RNAs to UV-irradiated chromatin (Fig. 1C-E, 2 E, F, and 3A, B).

In the next step, we continued to address whether the appearance of m^6^A RNA at UV-damaged chromatin is mediated via METTL3 methyltransferase. Recently, we showed that the level of METLL3 and METTL14 at DNA lesions is stable, which can be sufficient for DDR-related RNA methylation on 6-adenosine [27]. From this view, we additionally tested selective METTL3 inhibitor (STM2457) [51], and we observed a reduced level of m^6^A RNAs at DNA lesions induced in STM2457-treated cells. The DDR-specific inhibitory effect caused by the STM2457 compound was dependent on its concentration. In this case, we detected the most significant inhibitory effect for 2 µM concentration of STM2457 at 5–20 minutes after microirradiation (Fig. 3C-E). For this type of analysis, we tested the concentration and time of the treatment as recommended in Yankovska et al. (2021) [51].

### PARP inhibitor increased γH2AX positivity in the whole cell nuclei, did not change the level of CPDs at locally induced DNA lesions, and abolished the positivity of XRCC1 and APE1 to microirradiated chromatin

**Figure 4.**
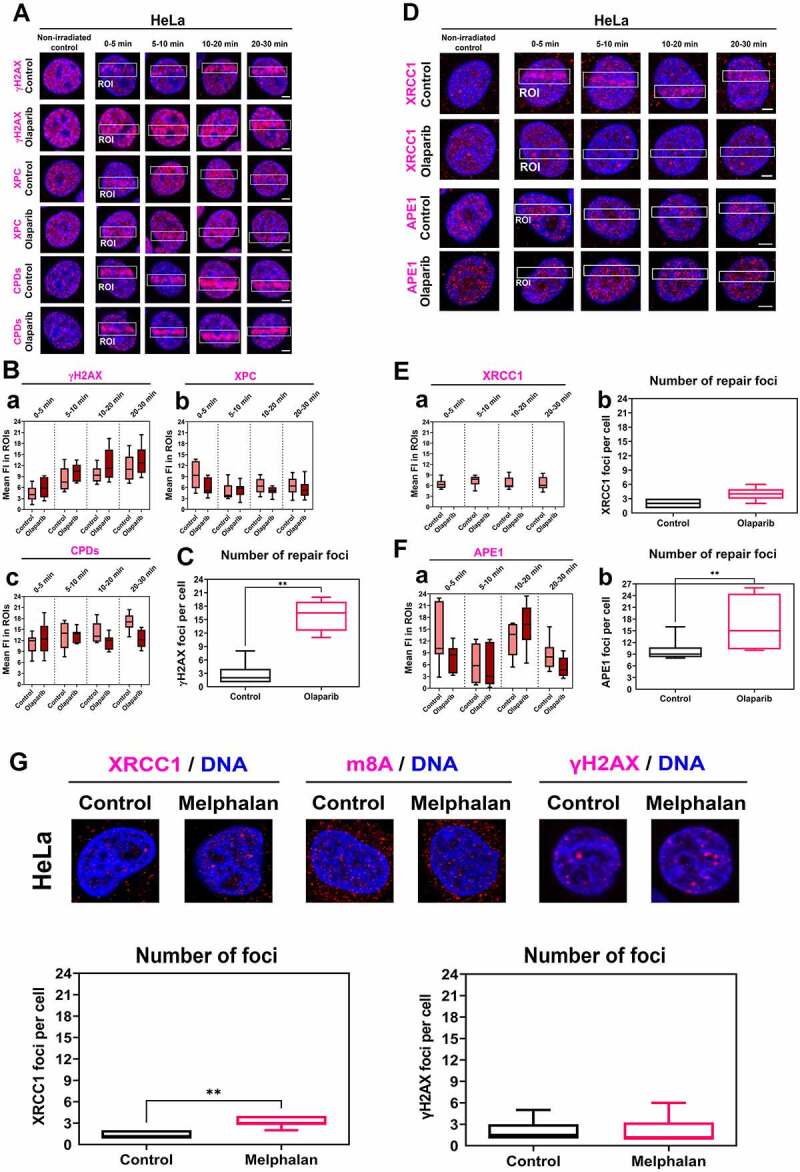
PARP inhibitor Olaparib abolished XRCC1 and APE1 recruitment to microirradiated chromatin, but no changes were observed in NER factors. (A) PAPRi did not change the levels of XPC and CPDs at DNA lesions, while Olaparib potentiates γH2AX positivity in the whole cell nuclei. Panel (B) shows quantification of fluorescence intensity of (a) γH2AX, (b) XPC, (c) CPDs in microirradiated chromatin. (C) PARPi causes an increase of γH2AX positive foci per cell. (D) PARPi prevents the recruitment of XRCC1 and APE1 to UVA-damaged chromatin but does not change the number of (E) XRCC1 positive foci. (F) The number of APE1 positive foci increased after PARP inhibition. Data are shown as the mean ± SE (standard error), and statistically significant differences are shown at p ≤ 0.01. (G) Treatment with an alkylating agent, Melphalan, increased the number of XRCC1-positive foci, but γH2AX positivity and m^8^A RNA level remained stable after Melphalan treatment.

We found that the PARP inhibitor, Olaparib, has no potential to change the levels of NER factors, including XPC and CPDs at DNA lesions. In contrast, Olaparib potentiates γH2AX positivity in the whole cell nuclei (Fig. 4A, Ba-c). PARPi also increased the number of γH2AX positive foci per cell (Fig. 4C). Olaparib prevents the accumulation of XRCC1 and APE1, factors of BER mechanism, to UVA-damaged chromatin (Fig. 4D). In detail, the level of XRCC1 was stable before and after microirradiation (Fig. 4Ea). At the same time, APE1 density in the irradiated genome was reduced 0–5 min postirradiation in Olaparib-treated cells (). The number of XRCC1-positive DNA repair foci was not affected by Olaparib treatment, but the number of APE1-positive repair foci increased after this treatment (Fig. 4Eb, Fb) we calculated repair foci larger than 1 µm).

We also studied the effect of an alkylating agent potentially inducing genomic lesions recognized by the BER mechanism. For this experiment, we used Melphalan, a clinically used cytostatic for multiple myeloma treatment [52], and we observed that Melphalan increases the number of XRCC1-positive DNA repair foci, but no changes were found when we compared m^8^A RNA nuclear level and γH2AX-positive foci in non-treated and Melphalan-treated cells (Fig. 4G).

### PARP inhibitor reduced the level of PARP1 in the cell nucleus and increased the number of 53BP1- and MDC1-positive DNA repair foci

**Figure 5.**
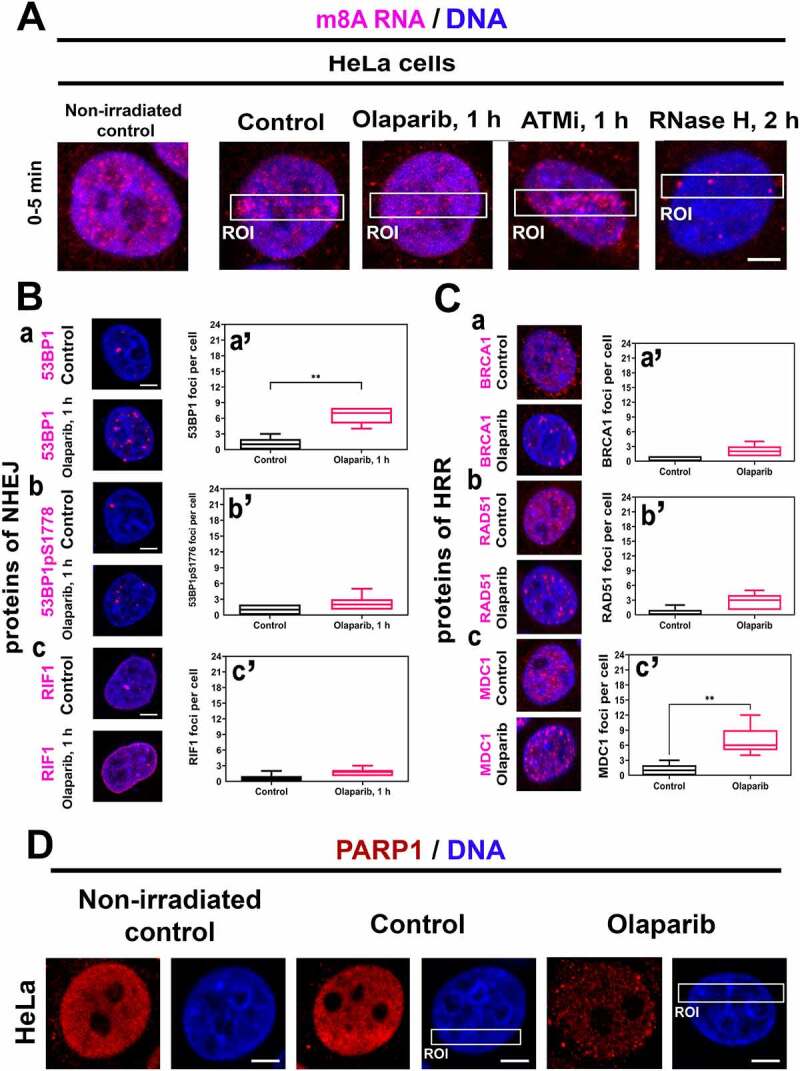
PARP inhibitor affects specific DNA repair foci and erases PARP1 positivity in microirradiated chromatin. (A) One hour after the cell treatment by PARPi, there was a low m^8^A RNA positivity at DNA lesions, and RNase H reduced the m^8^A RNA abundance in the whole genome. ATMi did not change the m^8^A RNA level in microirradiated chromatin. (B-C) PARPi significantly changed the number of (Ba, a’) 53BP1-, and (Cc, c’) MDC1-positive foci but did not change the number of (Bb, b’) 53BP1pS1778-, (Bc, c’) RIF1-, (Ca, a’) BRCA, (Cb, b’) RAD51-positive foci. Data are shown as the mean ± SE (standard error). Statistically, significant differences are shown at p ≤ 0.01. (D) PARP inhibitor reduced the level of PARP1 in both the whole genome and microirradiated genomic regions (ROI).

Because PARP inhibitor Olaparib abrogated positivity of m^8^A RNA, XRCC1, and APE1 at DNA lesions (Fig. 1Ci and 4D-F), we have additionally analysed if PARPi affects factors of non-homologous end joining (NHEJ) and homologous recombination repair. We also tested whether RNAse H treatment affects the level of m^8^A RNA at microirradiated chromatin. In this case, in Olaparib treated cells, m^8^A RNA was barely detectable, similarly to in the cells affected by RNAse H (Fig. 5A). An inhibitor of ATM did not prevent m^8^A RNA accumulation at microirradiated chromatin (Fig. 5A). In non-treated and Olaparib treated cells, we also calculated the number of DNA repair foci, positive on 53BP1, phosphorylated form of 53BP1, RIF1, BRCA1, RAD51, and MDC1 proteins (Fig. 5 Ba-c, Ba’-c’, Ca-c, Ca’-c’). After Olaparib treatment, the number of DNA repair foci, positive on 53BP1pS1778, RIF1, BRCA1, and RAD51 was not changed (Fig. 5 Bb’, c’; Ca’, b’). An exception was the increased number of 53BP1- and MDC1-positive repair foci, which appeared after Olaparib treatment (Fig. 5 Ba’, Cc’).

In addition to the above-mentioned observations, we verified if the PARP1 protein is recruited to UV-induced DNA lesions. We found an increased PARP1 level after microirradiation, and significantly, PARPi completely reduced PARP1 positivity in the whole genome, and in these cells, PARP1 did not recruit to microirradiated chromatin (Fig. 5D).

### m^8^A RNA interacted with DNA and γH2AX, while the highest FLIM-FRET efficiency was observed for m^6^A RNA and the XRCC1 protein

**Figure 6.**
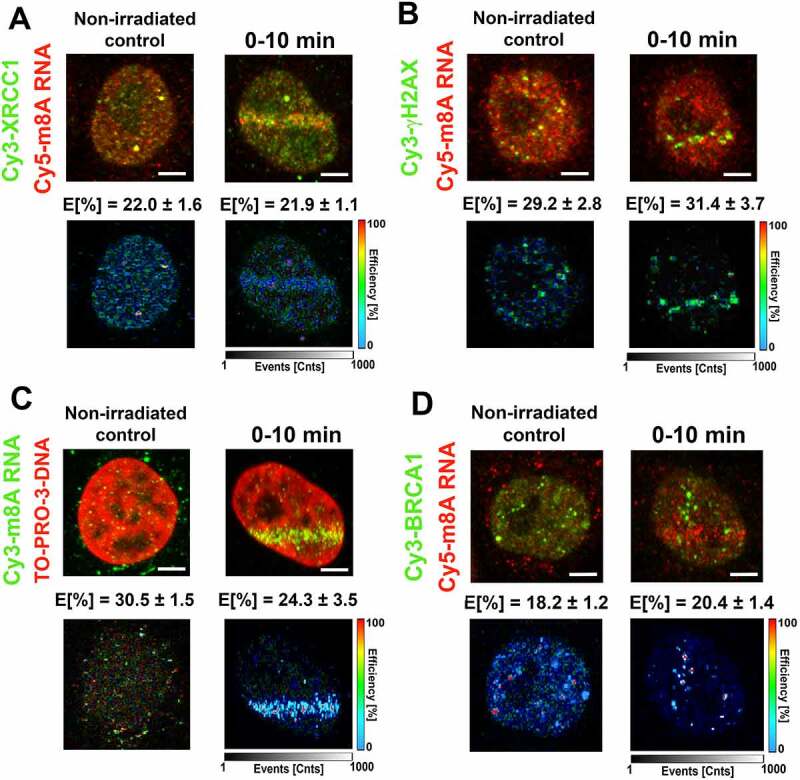
m^8^A RNA interacts with γH2AX and DNA. FLIM-FRET analysis showed a weak interaction between (A) m^8^A RNA and the XRCC1 protein but a significant interaction between (B) m^8^A RNA and γH2AX or (C) m^8^A RNA and DNA. (D) The FLIM-FRET efficiency for m^8^A RNA and BRCA1 was on the cut-off level, which is ~ 20%.

Using FLIM-FRET analysis, we studied a degree of interaction between m^8^A RNA (m^6^A RNA) and XRCC1, a protein that, similarly to m^8^A RNA or m^6^A RNA, was recruited to DNA lesions in microirradiated cells. Significantly, the treatment by PARP inhibitor abolished the accumulation of m^8^A RNA, m^6^A RNA, and XRCC1 in the microirradiated genome (Fig. 1Ai; 3A, B; 4D). When studying interaction properties, in the case of m^8^A RNA and XRCC1, we have observed the FLIM-FRET efficiency on the so-called cut-off level that we established at 20% [53] (Fig. 6A). However, we found a significant FLIM-FRET efficiency when we analysed m^8^A RNA interaction with γH2AX or m^8^A RNA binding to DNA. In these cases, FLIM-FRET efficiency was approximately 30%. Significantly, UV-microirradiation did not change the protein-m^8^A RNA or m^8^A RNA-DNA interaction properties (Fig. 6A-D).

**Figure 7.**
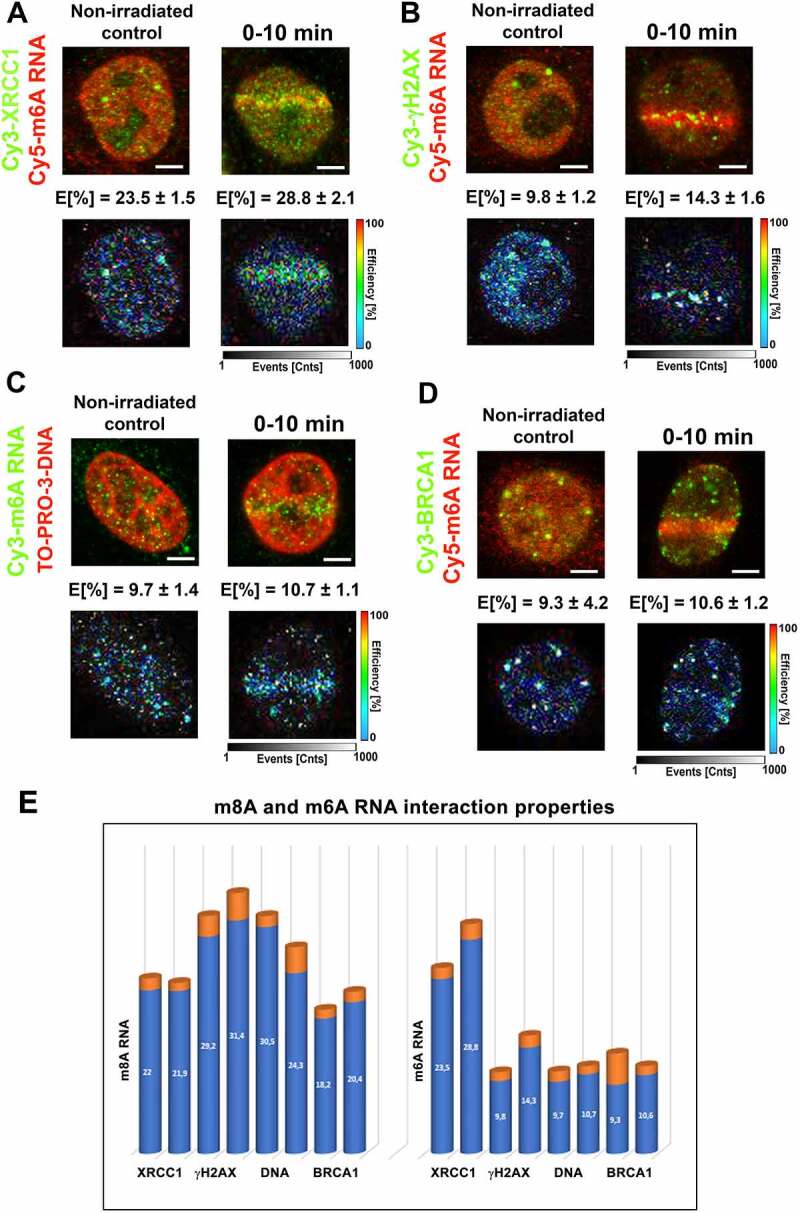
m^6^A RNA did not interact with γH2AX, DNA, and BRCA1. FLIM-FRET analysis showed a strong interaction between (A) m^6^A RNA and XRCC1, but no interaction was shown when analysing (B) m^6^A RNA and γH2AX or (C) m^6^A RNA and DNA or (D) m^6^A RNA and the BRCA1 protein. (E) Comparison of interaction properties in the following interaction partners: m^8^A RNA-XRCC1; m^8^A RNA-γH2AX, m^8^A RNA- DNA, and m^8^A RNA-BRCA1 and m^6^A RNA-XRCC1; m^6^A RNA-γH2AX, m^6^A RNA- DNA, and m^6^A RNA-BRCA1.

Surprisingly, in the case of m^6^A RNA, we have observed the highest FLIM-FRET efficiency for m^6^A RNA and XRCC1 (~28% in microirradiated cells); however, no interaction we found for m^6^A RNA and γH2AX; m^6^A RNA and DNA, or m^6^A RNA and BRCA1 (Fig. 7A-D). For comparison of interaction properties studied here, see in Fig. 7E.

In the case of m^8^A RNA (m^6^A RNA) and its interaction with the BRCA1 protein, FLIM-FRET efficiency was ~20% (~10%) (Fig. 6D, 7D). For the explanation, we tested an interaction between m^6^A RNA (or m^8^A RNA) with BRCA1 due to the observation published by Zhang et al. (2020) [31] or San Martin Alonso and Noordermeer (2021) [54], showing that the BRCA1 protein is recruited to m^6^A-modified R-loops, associated with DNA breaks. According to these authors, this type of RNA modification is essential for recognizing R-loops by the BRCA1 protein. To this point, we have to mention that the kinetics of m^6^A /m^8^A RNAs and the BRCA1 protein at DNA lesions is distinct: methylated RNAs appear at damaged chromatin 0–10 min after microirradiation (Fig. 1Ca, 3A, B), while BRCA1 recruits to the lesions 20 min post-irradiation [55]. Summarizing FLIM-FRET data in Fig. 7E, it is evident that the highest degree of interaction is between m^8^A RNA and γH2AX or DNA, and also m^6^A RNA significantly interacted with XRCC1.

Together, our data support the existence of m^6^A/m^8^A RNA-DNA hybrid loops. In such hybrid structures, DNA interacts with RNA via 8-methyladenosine, and loops are likely stabilized by the interaction between m^8^A RNA and γH2AX (Fig. 6B,). Moreover, both m^6^A RNA and m^8^A RNA interact with the XRCC1 protein, a member of the BER mechanism.

### The level of 5-hydroxymethylation (5hmC) and 5-carboxymethylation (5caC) was increased at locally microirradiated chromatin

**Figure 8.**
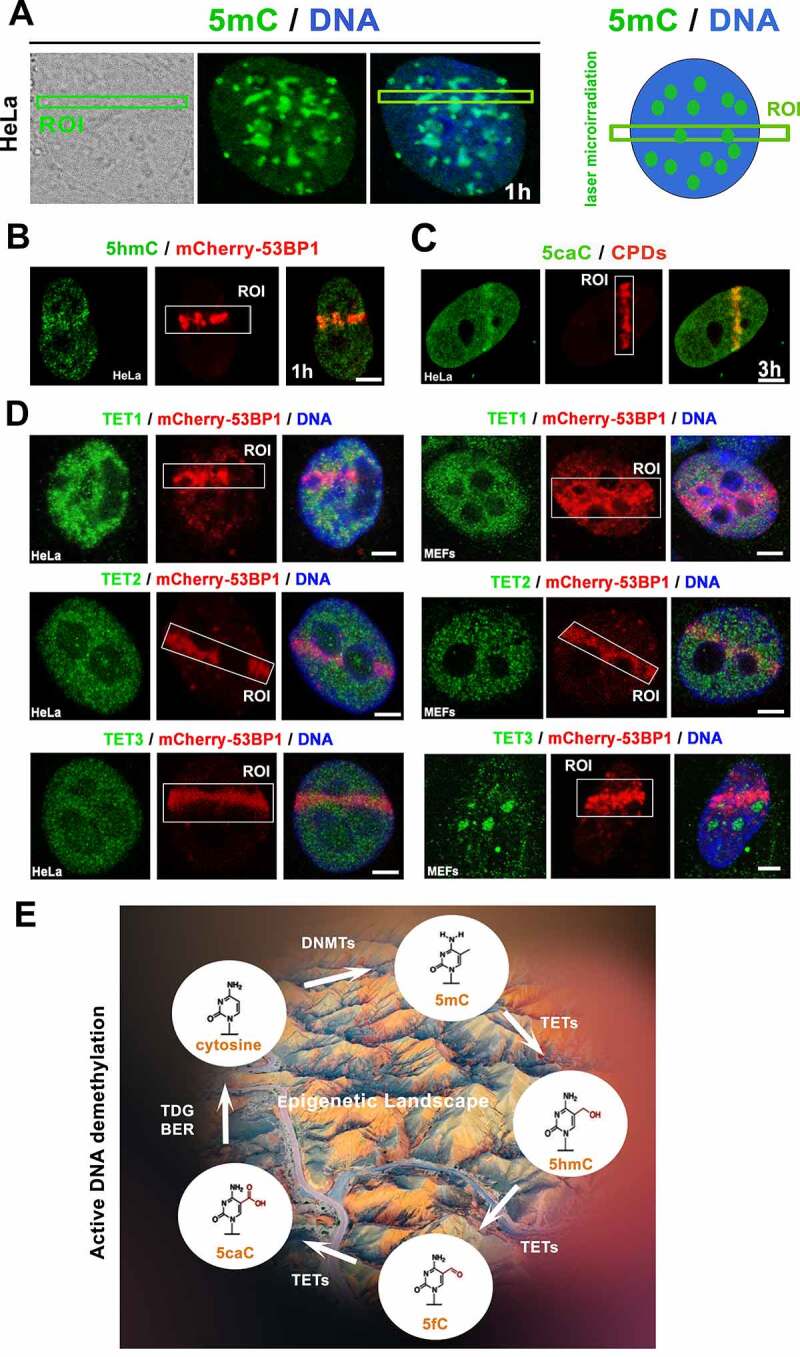
The active DNA demethylation in microirradiated chromatin. (A) nuclear distribution pattern of 5mC was studied in the microirradiated genome. (B) The level of 5hmC at locally induced DNA lesion is shown, similarly to (C) the level of 5caC. (D) TET1, TET2, and TET3 protein levels were studied in DNA lesions induced by UV laser in HeLa and MEFs cells. Scale bars show 4 µm. (E) Illustration of DNA demethylation processes.

In cells exposed to microirradiation, we observed that the level of 5-methyl cytosine (5mC) is relatively high but stable (Fig. 8A), but the density of 5hmC (5-hydroxymethyl cytosine) was increased in microirradiated chromatin (Fig. 8B). Most significantly, the level of 5-caC (5-carboxymethylation in DNA) increased in the microirradiated genomic region (Fig. 8C; note: density of 5hmC at microirradiated ROI is weaker than the level of 5caC, see Fig. 8B,). Interestingly to this observation, when the cell populations were exposed to UV light, the level of Ten-Eleven Translocation enzymes 1–3 (TET1, TET2, and TET3, responsible for the active DNA demethylation) was relatively stable (Fig. 8D). The stable level of these DNA demethylating proteins could be sufficient for the active DNA demethylation (Fig. 8E) appearing in the microirradiated genome as the later epigenetic event of DDR (Fig. 8B,).

## Discussion

Based on recently published data, it is evident that not only DNA but also RNAs must cope with genome injury. From this view, a fundamental observation of Xiang et al. (2017) [28] showed that m^6^A modified RNA and RNA-metabolizing proteins contribute to UV-induced DNA damage response. Also, Yu et al. (2021) [56] showed that the METTL3-METTL14 complex, similarly to m^6^A RNA nuclear reader YTHDC1, contributes to DNA repair. We observed the same for m^8^A RNAs, which appeared at DNA lesions in a PARP-dependent manner (Fig. 1Ci, D, E). From this view, Zhang et al. (2017) [31] suggested the existence of a new PARP- and METTL3/METTL14-dependent non-canonical m^6^A RNA-mediated DNA repair pathway recognizing UV damaged chromatin. However, Caron et al. (2019) [57] showed that PARP1 regulates DNA end resection of DSBs. These authors revealed that PARP1 protects DNA ends from nucleolytic degradation, but when PARP1 was depleted, it induced DNA hyper-resection due to weak DNA strand protection by the Ku80 factor. After PARP inhibition, the function of RIF1 and 53BP1, the proteins of the NHEJ, was abolished at DSB sites. Together, the above-mentioned observations show that m^6^A RNA and m^8^A RNA, in parallel with METTL3/METTL14 methyltransferases, are required for the repair of damaged chromatin [27,28]. However, the exact repair pathway, mediated via modified RNAs, is not fully elucidated.

In this case, an essential structural phenomenon represents hybrid DNA-RNA loops in which m^6^A RNA is considered as a template for homologous recombination repair (HRR) [58,59]. Also, m^6^A RNA readers, like YTHDF2 or YTHDC1, can recognize m^6^A RNA in R-loops, and these DDR-related conformation changes in chromatin could be responsible for the efficient DNA repair. Several authors suggested that R-loops at DNA lesions act positively and negatively, especially in homologous recombination repair [29, 32–36, 60,611]. Interestingly, R-loops in the vicinity of DSB sites can recruit factors of HRR, including RAD52 and BRCA1, in turn counteracting the anti-resection activity of the Shieldin complex, consisting of C20orf196 (also known as SHLD1), FAM35A (SHLD2), CTC-534A2.2 (SHLD3) and REV7 proteins recognizing DSB sites and working in 53BP1- and RIF1-mediated NHEJ repair pathway [62]. Recently, Abakir et al. (2020) [63] showed that m^6^A RNA is responsible for R-loops stability. Depletion of YTHDF2 caused an increased incidence of DSB sites. On the other hand, our data imply that m^6^A and m^8^A RNAs are involved in the repair of DNA lesions, primarily via a BER mechanism. We showed that the BER scaffold protein, XRCC1, in parallel with m^6^A and m^8^A RNAs, recruits to UV-induced DNA lesions in a PARP-dependent manner (Fig. 1A, 1, 1, 3 3-C, , 4D). PARP-dependence of XRCC1 function has also been reported by, for example, Adamowicz et al. (2021) [64]. Also, the role of XRCC1 and PARP in the direct SSB repair was shown by Strom et al. (2010) [43]. In addition to these observations, we show the most pronounced interaction between the XRCC1 protein and m^8^A RNA, or XRCC1 and m^6^A RNA, in which FLIM-FRET efficiency was strengthened after UV microirradiation (Fig. 6A and 7A). From this view, it is essential to mention that UV light induces CPDs that are recognized by NER factors [65]. Therefore, the exact role of XRCC1 and modified RNAs is not clear.

It must also be considered that the formation of hybrid RNA-DNA loops needs end-resection in S-phase. Thus, this process preferentially should proceed in the S phases of the cell cycle, but we have observed both m^6^A RNA and m^8^A RNA positivity at DNA lesions induced in the G1, S, and G2 phases of the cell cycle (Fig. 2A-D). Due to the functioning of modified RNAs during all phases of interphase and m^6^A/m^8^A RNA interaction with XRCC1, our results imply that m^6^A and m^8^A-modified RNAs rather contribute to the BER repair pathway.

In the literature, there is information about the appearance of R-loops in the RNAP II (RNA polymerase II) pause sites close to transcription termination sites [66,67]. Therefore, according to these data, the formation of methylated R-loops should be linked to the Transcription-Coupled Nucleotide Excision Repair (TC-NER) mechanism. In this case, an accumulation of the phosphorylated form of RNAP II is expected at UV-induced DNA lesions. However, we did not observe such a phenomenon [68]. Moreover, NER factors like CPDs and XPC were not affected by PARP inhibition ()(Fig. 4A). Together, in our experimental system, it seems likely that preferentially non-coding RNAs, but not mRNA, are components of R-loops in the irradiated genome. As Michelini et al. (2017) [69] or Vagbøa and Geir Slupphaug (2020) [59] showed, damage-induced long non-coding RNAs (dilncRNAs) or miRNAs can participate in DDR. Also, we must consider that these RNAs can be damaged by radiation; thus, their modifications could also be markers of RNA injury.

Here, we also find that the active DNA demethylation proceeds at DNA lesions as a later step of DDR. Especially, a high level of (5caC) was observed in damaged chromatin (Fig. 8B, C). This observation fits well with the data published by Zhu (2009) [70], demonstrating in *Arabidopsis thaliana* that a subfamily of DNA glycosylases promotes DNA demethylation through a base excision repair mechanism. Our observation suggests that active DNA demethylation also works in human cells via a base excision repair process activated by DNA damage. This nuclear event is mediated by TET enzymes (Fig. 8D) oxidizing 5-methylcytosines (5mC) to 5-hydroxymethylcytosine (5hmC), formyl cytosine (5fC), and 5-carboxylcytosine (5caC) in a Fe(II) and alpha-ketoglutarate (α-KG)-dependent manner [71]. Here, we observe an identical density of TET proteins before and after microirradiation, which did not exclude the possible functioning of these demethylating enzymes in the DDR mechanism studied (Fig. 8D).

Together, we showed and confirmed that the appearance of m^8^A RNA and m^6^A RNA at DNA lesions is PARP dependent (Fig. 1Ci, 3A, B). Due to a pronounced interaction between modified RNAs and XRCC1 and active DNA demethylation at the irradiated genome, we suggest that RNA modifications play a role in the BER mechanism, or alternatively, there is an existence of a non-canonical RNA-mediated DNA repair pathway, as suggested by Zhang et al. (2017) [45]. We also consider that R-loops are formed at DNA damage sites [72], showing that modified RNAs on m^6^A and m^8^A are essential for stabilizing DNA lesions. We also consider that UV-irradiation induces not only SSBs but also DSBs as the secondary structures, or UV irradiation can induce various types of DNA lesions. Thus, we observed a significant interaction between a DSB marker γH2AX and m^8^A RNAs (Fig. 6B). Suggested mechanism here shows a reciprocal link between epigenetic and epitranscriptomic events regulating DNA damage repair.

## Materials and methods

### Cell cultivation and treatment

The human cervix adenocarcinoma (HeLa) cell line (ATCC® CCL-2TM, ATCC, UK) was cultivated in EMEM (Eagle’s Minimum Essential Medium, Merck, Germany) supplemented with 10% foetal calf serum (FCS) and the appropriate antibiotics. Mouse embryonic fibroblasts (MEFs) are a gift from Prof. Thomas Jenuwein, Max Planck Institute of Immunology and Epigenetics, Freiburg, Germany). MEF cells were cultivated in DMEM (Dulbecco’s modified Eagle’s medium, Merck, Germany) supplemented with 10% FCS, β-mercaptoethanol (#31350-010, Thermo Fisher Scientific, USA), nonessential amino acids (100× NEAA; #1140-035, Thermo Fisher Scientific, USA), sodium pyruvate (#11360-039, Thermo Fisher Scientific, USA), 1.5 g of NaHCO_3_ (#S5761, Merck, Germany), and appropriate antibiotics at 37°C in a humidified atmosphere containing 5% CO_2_ (also see [73]).

To study the cell cycle-dependent recruitment of proteins to DNA lesions, we used HeLa-FUCCI cells expressing RFP-Cdt1 in the G1 phase and GFP-geminin in the S/G2/M phases previously been described in detail [74] (Life Technologies; http://www.lifetechnologies.com). HeLa-FUCCI cells were cultivated in Dulbecco’s modified Eagle’s medium supplemented with 10% FCS and appropriate antibiotics at 37°C in a humidified atmosphere containing 5% CO_2_.

The HeLa cells were treated with several different inhibitors listed in Table 1. This table summarizes the used compounds and their final concentration. It also contains the time of treatment duration and a short description of the main inhibition targets.

**Table 1.** Chemical compounds used in DNA repair studies.

Compound	Abbreviation	Final concentration/time	Catalogue number/producer	Description and reference
A196	A196i	5 μM/24 h	#SML1565/ Merck, Germany	SUV420H1/H2 inhibitor that inhibits the di- and trimethylation of H4K20me [46]
Actinomycin D	ActD	0.5 μg.ml^−1^/2 h	#A9415/ Merck, Germany	Inhibits mRNA transcription [65]
α-Amanitin	α-Amanitin	2 μg.ml^−1^/2 h	#A2263/ Merck, Germany	Inhibits eukaryotic RNA polymerase II and III and inhibits mammalian protein synthesis [75]
KU-55933	ATMi	16 μM/1 h or 24 h	#S1570/ Selleckchem, Germany	Inhibitor of the ATM kinase [76]
5-aza-2’-deoxycytidine	5-AzadC	10 μM/48 h	#A3656/ Merck, Germany	Inhibits DNA methyltransferase activity and causes DNA demethylation or hemi-demethylation [77]
Suberoylanilide hydroxamic acid	SAHA	15 μM/2 h	#SML0061/ Merck, Germany	Inhibits class I and class II HDACs [87]
Trichostatin A	TSA	100 nM/24 h	#T8552/ Merck, Germany	Inhibits histone deacetylase and results in histone hyperacetylation that leads to chromatin relaxation and modulation of gene expression [78]
Olaparib	PARPi	10 μM/1 h or24 h	#S1060/ Selleckchem, Germany	Induces significant autophagy that is associated with mitophagy in cells with BRCA mutations [79]
STM2457	METTL3i	2 µM or 4 µM/ h	A generous gift from Dr. Angus Lauder (STORM Therapeutics, UK)	Inhibits METTL3 methyltransferase [51]
RNAse H	RNase H	0.05 U. ml^−1^/2 h at 37°C	#EN0201, ThermoFisher Scientific, USA	Specifically degrades the RNA strand in RNA-DNA hybrids [86]
Melphalan	Melphalan	30 μM/24 h	#S8266/ Selleckchem, Germany	Alters the guanine via alkylation, causes linkages between strands of DNA, and inhibits DNA synthesis and RNA synthesis [80]

### Local micro‑irradiation of the genome and analysis of DNA lesions with laser scanning confocal microscopy

For local laser microirradiation, cells were seeded on uncoated, γ-irradiated, gridded microscope dishes (#81166, Ibidi, USA). At 50% confluence, cells were either transiently transfected with mCherry-tagged 53BP1 (mCherry-BP1-2 pLPC-Puro; a fragment of human 53BP1, aa 1220–1711; #19835, Addgene, USA [81],) or were treated with the list of inhibitors see Table 1 and/or pre-sensitized with 10 μM 5-Bromo-2′-deoxyuridine (BrdU; #11296736001, Merck, Germany) for 16–18 h and Hoechst 33,342 solution to final concentration 20 nM (#62249, Thermo Fischer Scientific, USA) see [82–84].

The mCherry-53BP1 plasmid’s DNA was amplified in chemically competent *E. coli* DH5α bacteria, and DNA was isolated using Qiagen Plasmid Maxi Kit (#121693, QIAGEN, Bio-Consult, the Czech Republic). Transfections were performed using METAFECTENE^TM^PRO reagent (#T040-2.0, Biontex Laboratories GmbH, Germany), see [55].

The cells were irradiated using a TCS SP5-X confocal microscope system (Leica, Germany) equipped with the 355-nm and 405-nm laser and 63x oil objective (HCX PL APO, lambda blue) with a numerical aperture (NA) = 1.4. The irradiation conditions were optimized according to Refs. [28,55,85].

The cells were maintained under optimal cultivation conditions in an incubation chamber (EMBL, Germany) at 37°C, and the cell culture hood was supplemented with 5% CO_2_. Image acquisition for the induction of DSBs was performed with the following settings: 1024 × 1024 pixels, 400 Hz, bidirectional mode, zoom 2. Micro-irradiated cells were monitored for 30 min or at the exact time points of 1 h and 3 hours. Immunofluorescence staining was performed at the following intervals (in minutes): 0–5, 5–10, 10–20, 20–30, or in the time points 60 min and 180 min. After the staining procedure, identical irradiated cells were found on microscope slides using the registered coordinates at gridded microscopic dishes. We quantified the relative fluorescence intensity of either Alexa Fluor 594 dye in HeLa cells or Cy5 dye in HeLa-FUCCI cells, which were used to visualize the protein accumulations in the regions of interest (ROIs). We used Leica LAS X software to analyse fluorescence intensity or the number of foci per cell. The statistical analysis was performed using GraphPad Prism 9 software (USA) and the nonparametric Mann–Whitney *U*-test. The asterisks in the figures represent statistical significance with a p-value ≤0.01. The data are presented as the mean ± standard error (SE), representing average values from 40 to 60 evaluated cell nuclei per each time point.

### Immunofluorescence staining

Immunofluorescence was modified following [26]. The cells were fixed in 4% formaldehyde (PFA; #AAJ19943K2, Fisher Scientific, USA) for 10 min at room temperature (RT), permeabilized with 0.2% Triton X-100 (#194854, MP Biomedicals, USA) for 8 min, and 0.1% saponin (#S7900, Merck, Germany) for 13 min. In the next step, the microscopic dishes were washed twice in phosphate buffer saline (PBS) for 15 min. We used 10% goat serum (#G9023, Merck, Germany) dissolved in 1x PBS-Triton X-100 (0.1%) as a blocking solution.

The samples were incubated for 1 h at room temperature and then washed in 1x PBS for 15 min. For immunofluorescence analysis, the following antibodies were used: anti-m^8^A (#ab211498, Abcam, UK), anti-γH2AX (#ab2893, Abcam, UK), anti-XRCC1 (#sc56254, Santa Cruz Biotechnology Inc., USA), anti-XPC (#sc74410, Santa Cruz Biotechnology Inc., USA), anti-BRCA1 (#sc6954, Santa Cruz Biotechnolgy Inc., USA), anti-RAD51 (ab88572, Abcam, UK), anti-MDC1 (#NBP2-12,890, Novus Biological, UK), anti-53BP1 (#MAB3802, Merck, Germany), anti-53BP1pS1778 (#S2675, Cell Signalling Technology, USA), anti-RIF1 (#GTX131889, GeneTex, Taiwan), anti-PARP1 (#A2432, ABclonal, USA), and anti-APE1 (#10519S, Cell Signalling Technology, USA). The indicated primary antibodies were diluted at 1:100 in blocking buffer and incubated at 4°C overnight.

For m^6^A, 5-methylcytosine (5mC), 5-hydroxymethylcytosine (5hmC), 5-carboxylcytosine (5caC), TET1, TET2, and TET3 proteins, the immunostaining was performed following [28]. Briefly, the cells were fixed in 4% PFA for 10 min at RT, then permeabilized in 0.25% Triton X-100 for 30 min. After washing with PBS. As a blocking solution, we used a PBS containing 1% Bovine Serum Albumin (BSA; #A2153, Meck, Germany), 10% Foetal Bovine Serum (FBS; FB-1090/500, BioTech, the Czech Republic), and 0.25% Triton X-100 for 60 min at RT. The following primary antibodies were used: anti-m [6]A (#202003, Synaptic System, Germany), anti-5mC (#ab10805, Abcam, UK) anti-5hmC (#39999, Active Motif, Germany), anti-5caC (#61225, Active Motif, Germany), anti-TET1 (#1506, ABclonal, USA), anti-TET2 (#16273, ABclonal, USA) and anti-TET3 (#7612, ABclonal, USA). The dilution concentration was 1:100 in blocking buffer, and incubation was at 4°C overnight.

The following secondary antibodies were used: Alexa Fluor 488-conjugated donkey anti-mouse (#A21202, Thermo Fisher Scientific, USA), Alexa Fluor 488-conjugated goat anti-rabbit (#ab150077, Abcam, UK), Alexa Fluor 594-conjugated goat anti-mouse (#A11032, ThermoFisher Scientific, USA), Alexa Fluor 594-conjugated goat anti-rabbit (#A11037, ThermoFisher Scientific, USA), and goat anti-rabbit Cy5 (#ab6564, Abcam, UK). The secondary antibodies were diluted at 1:200, and the incubation time was 60 min at RT.

For Cyclobutane Pyrimidine Dimers (CPDs) staining, the cells were fixed in 4% PFA for 10 min at RT, then permeabilized in 0.5% Triton X-100 for 5 min on ice, followed by denaturation with 2 M HCl for 30 min at RT. After washing with PBS, cells were blocked in a solution containing 20% FBS dissolved in 1x PBS for 60 min at 37°C. After the washing step (five times in 1x PBS), cells were incubated with primary antibody anti-CPDs (#NMDND001, Cosmo Bio Co., Ltd., Japan) for 60 min at 37°C. The cells were rewashed twice with 1x PBS and incubated with a secondary antibody Alexa Fluor 594-conjugated goat anti-mouse (#A11032, ThermoFisher Scientific, USA; dilution 1:100) for 60 minutes at 37°C.

For RNase H treatment, we adapted a protocol according to Kotsantis et al. (2016) [86] and Xiang et al. (2017) [28]. Cells were washed in 1% Triton X-100 and then fixed in 4% formaldehyde for 10 min at room temperature. Followed by permeabilization with 0.3% Triton X-100 in 1x PBS for 30 minutes and washed in 1x PBS for 15 minutes at RT. RNase H (#EN0201, ThermoFisher Scientific, USA) was used at 0.05 U/ml for 2 hours at 37°C. The microscopic dishes were washed 1x PBS for 15 min and blocked with 3% BSA, 10% FCS and 0.1% Triton X-100 in dissolved in 1x PBS for 60 min at RT. The following primary and secondary antibodies were used: anti-m [8]A (#ab211498, Abcam, UK) and Alexa Fluor 594-conjugated goat anti-rabbit (#A11037, ThermoFisher Scientific, USA).

We incubated samples with antibodies dilution buffers without the primary antibodies as negative controls. We did not observe signals due to the non-specific binding of secondary antibodies.

A contour of cell nuclei (condensed chromatin) was visualized by the use of 4′,6-diamidino-2-phenylindole (DAPI; #D9542, Merck, Germany), dissolved in Vectashield (#H-1000, Vector Laboratories, USA).

We acquired images with Leica TCS SP8X SMD confocal microscope (Leica Microsystem, Germany), equipped with HC PL APO 63×/ 1.4 oil CS2 objective. Image acquisition was performed using a white light laser (WLL) with the following parameters: 1024 × 1024 pixel resolution, 400 Hz, bidirectional mode, and zoom 2. As described above, we used Leica Application Suite (LAS X) software for immunofluorescence analysis.

### FLIM-FRET technique

Fluorescence Lifetime Image (FLIM) microscopy combined with Förster Resonance Energy Transfer (FRET) was performed following [87]. Using this method, we studied the interactions between XRCC1 protein (donor) and m^8^A RNA or m^6^A RNA (acceptor), γH2AX (donor), and m^8^A RNA (acceptor), and m^8^A RNA/m^6^A RNA (donor) and DNA (acceptor). The protein–protein interactions were studied in fixed, immunostained samples with the use of the following primary antibodies: anti-m [8]A (#ab211498, Abcam, UK), anti-m [6]A (#202003, Synaptic System, Germany), anti-XRCC1 (#sc56254, Santa Cruz Biotechnology Inc., USA), anti-BRCA1 (#sc6954, Santa Cruz Biotechnology Inc., USA) and anti-γH2AX (#05-636, Merck, Germany).

As secondary antibodies, we used goat anti-mouse Cy3 (#ab97035, Abcam, UK), goat anti-rabbit Cy3 (#A10520, Thermo Fisher Scientific, USA), goat anti-rabbit Cy5 (#ab6564, Abcam, UK), and TO-PRO-3 Iodide (#T3506, Thermo Fisher Scientific, USA).

The fluorophores are characterized according to their absorption and fluorescence properties; for example, the higher extinction coefficient (EC) leads to absorbing a more significant amount of light [88]. The specific characteristics of fluorophores used in our FLIM-FRET experiments were adopted from the webpage https://www.fpbase.org/fret/ and are summarized in Table 2.

**Table 2.** Specification of donors and acceptors used for FLIM-FRET experiments.

QY_Cy3_ (DONOR)	EC _Cy5_ (M^−1^cm^−1^)	QY_Cy5_ (ACCEPTOR)	J(λ) (*1e15 M^−1^cm^−1^nm^4^)	R_0_(Å)	R_0_×QY_Cy5_
0.15	250 000	0.30	7.6	49.73	14.92
QY_Cy3_ (DONOR)	EC _TO-PRO-3_ (M^−1^cm^−^[1])	QY _TO-PRO-3_ (ACCEPTOR)	J(λ) (*1e15 M^−1^cm^−[^1,m^4^^]^)	R_0_(Å)	R_0_×QY_Cy3_
0.15	150 000	0.65	3.9	44.49	28.92

QY = Quantum Yield; EC = Extinction Coefficient; J(λ) = Overlap Integral, $J\left(\lambda \right)=\overset{\propto }{\underset{0}{\int }}F_{D}\left(\lambda \right)\varepsilon_{A}\left(\lambda \right)\lambda^{4}d\lambda /\overset{\propto }{\underset{0}{\int }}F_{D}\left(\lambda \right)d\lambda$; R_0_ = Föster Radius, $R_{0}=0.211\sqrt[{6}]{\kappa^{2}n^{-4}Q_{D}J\left(\lambda \right)}$; n = refractive index and κ^2^ = orientation factor κ^2^ = 0.6667.

All samples were mounted in Vectashield (#H-1000, Vector Laboratories, USA). Measurement was performed using Leica TCS SP8 X SMD confocal microscope (Leica Microsystems GmbH, Germany), PicoHarp 300 module (PicoQuant GmbH, Germany), and HyD SMD detectors. For cell visualization, we used a 63× oil immersion objective of numerical aperture 1.4. We used the pulsed white-light laser (WLL) as the excitation source with a repetition rate of 20 MHz. We acquired at least 1500 photons/pixel at the resolution of 512 × 512 pixels. Results were analysed by SymPhoTime 64 software (PicoQuant GmbH, Germany), and FRET efficiency was calculated following [89,90]. Using the FLIM-FRET technique, we studied up to 40 cell nuclei for each experimental event.

### Western blot analysis

The western blot analysis was performed following [91]. Briefly, to achieve identical concentrations of total proteins, we measured protein concentration by µQuant spectrophotometer (BioTek, USA). Then, the proteins were separated using SDS polyacrylamide gel electrophoresis (SDS-PAGE), followed by transfer to polyvinylidene difluoride (PVDF) membranes. In the next step, the membranes were blocked in either 2% non-fat dry milk or 2% BSA for 2 h and then incubated overnight at 4°C with the following primary antibodies: anti-H4K20me2 (#NB21-2089, Novus Biological, UK), anti-H4K20me3 (#A4048, EpigenTek, USA), H3K9ac (#06-942, Merck, Germany) and anti-γH2AX (#05-636, Merck, Germany). Primary antibodies were diluted to 1:1000. Next day, after washing, the membranes were incubated with the following secondary antibodies: goat anti-rabbit IgG (#AP307P, Merck, Germany) and goat anti-mouse IgG1 (#sc-2060, Santa Cruz Biotechnology, USA). Secondary antibodies were diluted to 1:2000 in blocking solutions. The western blot data (density of bands) were measured by ImageJ software (NIH freeware).

### Quantitative image-based cytometry

The samples for quantitative image-based cytometry (QIBC) were prepared as describ [92]. Briefly, cells were pre-extracted with ice-cold CSK buffer (10 mM Hepes, pH 7.5, 100 mM, NaCl, 300 mM sucrose, 3 mM MgCl_2_) for 10 min on ice and then fixed with 4% buffered formaldehyde (#9713.1000, VWR Chemicals, USA) for 30 min at room temperature. Primary antibody against PCNA (human, dilution 1:1000, #2037, Immunoconcepts, USA) and secondary antibody Alexa Fluor 647-conjugated donkey anti-human (dilution 1:1000, #709-605-149, Jackson Immuno Research, USA) were diluted in DMEM containing 10% FBS. The incubation times were 90 and 30 minutes for the primary and secondary antibodies, respectively. The secondary antibody solution was supplemented with 0.5 µg/ml of DAPI. After staining, samples were washed three times in 1x PBS and twice in double distilled water, dried, and mounted into a Mowiol-based mounting medium (12 % Mowiol 4-88 (#81381, Merck, Czech Republic), 30 % glycerol, 0.12 M Tris-HCl pH 8.5).

Images were captured using a ScanR high-content screening station (Olympus, Czech Republic) equipped with IX83 inverted microscope, wide-field optics, UPLSAPO dry objective (20×, 0.75 NA), Lumencor Spectra X LED fluorescence light source, filters for wavelengths of DAPI, FITC, Cy3 and Cy5, and digital Hamamatsu ORCA-R2 CCD camera. Images were acquired and analyzed automatically using ScanR acquisition and analysis software (v3.3.0, Olympus). Data were then exported to TIBCO Spotfire software (v11.8.0, TIBCO, USA) for further analysis. At least 1000 cells were compared for each condition within one experimental data set.

        

## Data Availability

https://www.ibp.cz/en/research/departments/molecular-cytology-and-cytometry/research-profile

## Statistical analysis

For statistical analyses, we used GraphPad Prism 9 software (USA). If the data passed the normality test, the Student’s test was applied. If not, the Mann-Whitney U-test was used. The results of the statistical tests were mentioned directly in the figure legend.
